# Supplementary material for: Virulence Evolution: Thinking Outside of the Host
Source: Evol Appl. 2025 Aug 29;18(9):e70136. doi: 10.1111/eva.70136 (PMC12395352; doi:10.1111/eva.70136)
Supplement: Supplementary file 1 — Table S1. Parasite infectivity and infection severity in a new host. Table S2. Q p and Q e on infectivity and infection severity. Table S3. Virulence‐transmission decomposition. [file EVA-18-e70136-s001.docx]

**Supplementary information**

for " Virulence evolution: thinking outside of the host"

**Table S1.** Parasite infectivity and infection severity in a new host.

**Table S2.** *Qp* and *Qe* on infectivity and infection severity.

**Table S3.** Virulence-transmission decomposition.

**Table S1. Parasite infectivity and infection severity in a new host.**

| **Tested effect** |  |  |  |
| --- | --- | --- | --- |
| **Model 1a** - Proportion of females with spores | **χ2** | ***df*** | ***p*** |
| Treatment | 32.996 | 2 | **< 0.001** |
| Temperature | 77.557 | 1 | **< 0.001** |
| Time | 166.849 | 1 | **< 0.001** |
| Treatment: Temperature | 1.011 | 2 | 0.603 |
| Treatment: Time | 3.446 | 2 | 0.179 |
| Temperature: Time | 0.433 | 1 | 0.511 |
| Treatment: Temperature: Time | 1.867 | 2 | 0.393 |
|  |  |  |  |
| **Model 1b -** Spore load | **χ2** | ***df*** | ***p*** |
| Treatment | 135.307 | 2 | **< 0.001** |
| Temperature | 0.002 | 1 | 0.961 |
| Time | 14.228 | 1 | **< 0.001** |
| Treatment: Temperature | 0.814 | 2 | 0.666 |
| Treatment: Time | 45.506 | 2 | **< 0.001** |
| Temperature: Time | 4.459 | 1 | **0.035** |
| Treatment: Temperature: Time | 7.352 | 2 | **0.025** |
|  |  |  |  |

**Table S2. *Qp* and *Qe* on infectivity and infection severity.**

| **Tested effect** | |  | |  | |  |  |
| --- | --- | --- | --- | --- | --- | --- | --- |
| **Model 2c** - *Qp* on infectivity | | **χ2** | | ***df*** | | ***p*** |  |
| Treatment | | 11.286 | | 2 | | **0.004** |  |
| Multiple comparisons for Model 2a | |  | | ***t*-ratio** | | ***p*** |  |
| Early - Late | |  | | 3.106 | | **0.035** |  |
| Early - Stock | |  | | 0.444 | | 0.900 |  |
| Late - Stock | |  | | -2.662 | | 0.067 |  |
|  | |  | |  | |  |  |
| **Model 2d -** *Qp* on infection severity | | **χ2** | | ***df*** | | ***p*** |  |
| Treatment | | 9.022 | | 2 | | **0.011** |  |
| Multiple comparisons for Model 2b |  | | ***t*-ratio** | | ***p*** | | |
| Early - Late | |  | | 1.884 | | 0.205 |  |
| Early - Stock | |  | | -1.083 | | 0.550 |  |
| Late - Stock | |  | | -2.968 | | **0.043** |  |
|  | |  | |  | |  |  |
| **Model 2e** - *Qe* on infectivity | | **χ2** | | ***df*** | | ***p*** |  |
| Treatment | | 0.247 | | 2 | | 0.884 |  |
|  | |  | |  | |  |  |
| **Model 2f -** *Qe* on infection severity | | **χ2** | | ***df*** | | ***p*** |  |
| Treatment | | 1.809 | | 2 | | 0.405 |  |
|  | |  | |  | |  |  |

**Table S3. Virulence-transmission decomposition.**

| **Tested effect** |  |  |  |
| --- | --- | --- | --- |
| **Model 4a** - *Qp* on infectivity *vs.* virulence | **F** | ***df*** | ***p*** |
| log(Maximum hazard +1) | 12.934 | 1 | **< 0.001** |
|  |  |  |  |
| **Model 4b -** *Qp* on infection severity *vs.* virulence | **F** | ***df*** | ***p*** |
| log(Maximum hazard +1) | 4.351 | 1 | 0.057 |
|  |  |  |  |
| **Model 4c** - *Qe* on infectivity *vs.* virulence | **F** | ***df*** | ***p*** |
| log(Maximum hazard +1) | 0.464 | 1 | 0.508 |
|  |  |  |  |
| **Model 4d -** *Qe* on infection severity *vs.* virulence | **F** | ***df*** | ***p*** |
| log(Maximum hazard +1) | 0.155 | 1 | 0.701 |
|  |  |  |  |
